# Advances in Clinical Sample Preparation for Identification and Characterization of Bacterial Pathogens Using Metagenomics

**DOI:** 10.3389/fpubh.2018.00363

**Published:** 2018-12-12

**Authors:** Nathan L. Bachmann, Rebecca J. Rockett, Verlaine Joy Timms, Vitali Sintchenko

**Affiliations:** ^1^Sydney Medical School, University of Sydney, Camperdown, NSW, Australia; ^2^Centenary Institute, University of Sydney, Camperdown, NSW, Australia; ^3^Centre for Infectious Diseases and Microbiology–Public Health, Westmead Hospital, Sydney, NSW, Australia

**Keywords:** metagenomic, culture-independent genome sequencing, bacterial pathogens, *Mycobacterium tuberculosis*, public health

## Abstract

Whole genome sequencing (WGS) plays an increasing role in communicable disease control through high-resolution outbreak tracing, laboratory surveillance and diagnostics. However, WGS has traditionally relied on microbial culture in order to obtain pathogen specific DNA for sequencing. This has severely limited the application of whole genome sequencing on pathogens with fastidious culturing requirements. In addition, the widespread adoption of culture-independent diagnostic tests has reduced availability of cultured isolates for confirmatory testing and surveillance. These recent developments have created demand for the implementation of techniques enabling direct sequencing of microbial genomes in clinical samples without having to culture an isolate. However, sequencing of specific organisms from clinical samples can be affected by high levels of contaminating DNA from the host and other commensal microorganisms. Several methods have been introduced for selective lysis of host cells and/or separate specific organisms from a clinical sample. This review examines the different approaches for sample preparation that have been used in diagnostic and public health laboratories for metagenomic sequencing.

## Introduction

The ability to perform high-throughput whole genome sequencing (WGS) on infectious agents has revolutionized research into microbial genomics (1–3). WGS has become the method of choice for subtyping of pathogens with epidemic potential and investigating nosocomial transmission, foodborne disease outbreaks, and antibiotic resistance (4). Further, WGS has been extensively applied to investigate etiology, evolution and transmission of respiratory illnesses (5) and sexually transmitted diseases (6) as well as the acquisition and dissemination of antibiotic resistance in hospitals (7, 8). The ultimate resolution provided by WGS has led to its widespread use by public health facilities, such as Public Health England, the USA Centers for Disease Control and Prevention and others to investigate outbreaks of foodborne-associated salmonellosis (9), listeriosis (10) and Shiga-toxin producing *Escherichia coli* (11). The current status of culture-based WGS in clinical settings and sequencing platforms are reviewed recently elsewhere (12–14). However, the utility of WGS for communicable disease control is hampered by the reliance on the laboratory culture of the pathogen of interest.

Molecular-based culture independent diagnostics test (CIDT) methods offer significant advantages for diagnostic microbiology laboratories, the most important being a reduction in cost and an decrease in turn-around-time (15–17). The adoption of CIDT by diagnostic laboratories has led to significant increases in testing and diagnosis of enteric pathogens, but has reduced the number of isolates available for typing, resulting in notifications to public health registries without corresponding culture. As a direct result, our current laboratory surveillance systems are losing the capacity to detect clusters and identify the source of outbreaks and to recognize the emergence of high-risk or multi-drug resistant variants. The steady increase in CIDT-only notifications demands alternative methods for identification and characterization of bacterial pathogens with epidemic potential.

Metagenomics offers unbiased sequencing of all DNA in a clinical sample without culturing individual bacterial isolates [reviewed in (18)]. The applications of metagenomics have been largely discovery focused, probing the previously uncharacterized make up of environmental and human ecosystems and uncovering their unrecognized diversity (19). Furthermore, metagenomics has also been successful in identifying and characterizing emerging infectious agents (20). Metagenomic sequencing directly from clinical specimens eliminates the pathogen culturing step and could allow for comprehensive surveillance of antibiotic resistance and transmission dynamics. The application of metagenomics to diagnostics and outbreak tracing introduces many unique challenges regarding sample storage, DNA extraction, bioinformatics data analysis and reporting [reviewed in (21, 22)]. However, one of the largest barriers is to selectively target for microbial DNA in human samples. While bioinformatics tools can remove human reads, greater sequencing depth (which leads to an increase in cost) is required to obtain enough pathogen reads for identification of a causative agent and obtain information regarding resistance or strain type. This minireview will examine recent advances in techniques for overcoming this challenge, in particular how to deal with host and other contaminating DNA, with a specific focus on pathogens of public health importance.

## Metagenomic Sequencing For Public Health

The shotgun metagenomics has been applied for pathogen discovery or to uncover the etiology of an unrecognized infection (23, 24). Metagenomics has improved the diagnosis of central nervous system infections (24). Metagenomic will also been vital for understanding of other syndromes as unknown pathogens are estimated to be responsible for 20–40% of respiratory tract infections (25) and 40% of infections of the gastrointestinal tract (26). Currently, researchers have demonstrated that shotgun metagenomics has the equivalent sensitivity to diagnostic PCR-based methods, particularly when looking for pathogens in the gastrointestinal tract, respiratory system and urogenital tract (Table 1) (33, 34).

**Table 1:** Sample types and processing techniques for metagenomics of bacterial pathogens of public health importance.

**Niche/sample**	**Pathogen**	**Sample type**	**Sample challenge^*a*^**	**Methods used**	**WGS technologies evaluated**	**Expected or demonstrated added value**	**References**
Food borne pathogens	*Salmonella enterica*	Fecal	Homogenization	Direct metagenomics	Illumina HiSeq	Strain characterization and outbreak tracing	(27)
	*Shiga-toxin producing Escherichia coli*	Fecal	Homogenization	Direct metagenomics	Illumina HiSeq	Strain characterization and outbreak tracing	(11)
	*Listeria monocytogenes*	Fecal	Homogenization	Direct metagenomics	Illumina HiSeq	Strain characterization and outbreak tracing	(27)
	*Campylobacter* species	Fecal	Homogenization	Direct metagenomics	Illumina HiSeq	Strain characterization and outbreak tracing	(27)
Respiratory pathogens	*Mycobacterium tuberculosis*	Sputum	Liquification	Probe based capture, host cell lysis	Illumina HiSeq, MiSeq an Mini Ion	Detection and resistance profile	(28, 29)
	*Bordetella pertussis*	Nasopharyngeal aspirate	Low bacterial load/DNA yield	None published	None used	Detection and strain characterization	
	*Legionella pneumophila*	Sputum	Liquification/Low bacterial load/DNA yield	None published	None used	Detection and strain characterization	
	*Streptococcus pneumoniae*	Sputum/swab	Liquification	16S metagenomics	Illumina HiSeq	Detection and strain characterization	(30)
Sexually transmitted pathogens	*Chlamydia trachomatis*	Urine, swab	Low bacterial load/DNA yield	Probe based capture	Illumina HiSeq	Strain characterization	(31)
	*Mycoplasma genitalium*	Urine, swab	Low bacterial load/DNA yield	None published	None used	Detection and Resistance profile	
	*Treponema pallidum* (Syphilis)	Swab	Low bacterial load/DNA yield	None published	None used	Strain identification, subtyping and drug resistance	
	*Neisseria gonorrhoeae*	Urine, swab	NEBNext microbiome kit	Direct metagenomics	Ion Torrent PGM	Resistance profile	(32)

a *Specific challenges during DNA extraction that needs to be considered. Fecal samples will need to be homogenized with a tissue lyser or Qiagen's DNA Stool Mini Kit, while sputum samples will need to be treated with a decongest solution for liquification. Urine and swab samples will have challenges associated with DNA yield due to low cell count*.

The vast majority of sequencing reads produced during shotgun metagenomics are identified as human, which is on average 1000 times larger than the average bacterial genome. Direct shotgun metagenomics has limited utility in public health outbreak investigations due to the high cost of deep sequencing but it has been highly successful in diagnostics (32). As an example, metagenomics enabled the recognition of *Brucella* in cerebrospinal fluid (CSF) from a patient with partially treated meningitis where the causative agent could not be determined via traditional microbiological methods targeting common neurotropic pathogens (35). Although the CSF was positive for Epstein Barr virus and human herpesvirus 7, testing for bacterial pathogens including *Brucella* by serology and tuberculosis by culture were negative. When the patient's health failed to improve following antiviral treatment, the CSF was sent for metagenomic sequencing for a comprehensive identification of potential pathogens. Whole DNA extraction was performed on the sample, with half of extracted DNA been treated with turbo DNase followed by reverse transcription of the RNA to cDNA. The second half of the sample was treated with the NEBNext® Microbiome DNA Enrichment kit to enrich the microbial DNA. Both the enriched DNA and the cDNA were sequenced with Illumina HiSeq. From the raw data of the enriched DNA 0.0012% of reads corresponded to *Brucella melitensis*. It was noted that no *Brucella* reads were detected in the sequenced cDNA library. The patient was subsequently diagnosed with chronic neurobrucellosis and treated with doxycycline and rifampin with full resolution of symptoms in 2 weeks. This case demonstrates the utility of metagenomics sequencing as a supplementary test for notifiable conditions as *Brucella sp* was not originally cultured from CSF or blood cultures and the importance of microbial DNA enrichment as *Brucella* reads were also not found in the reserved transcribed RNA library.

## Processing of Clinical Samples

The success of metagenomics is dependent on quality and quantity of DNA extracted from a given specimen. Different specimen types (e.g., feces, sputum, tissue, urine, etc.) present unique and specific challenges reflecting their matrix and concentrations of the target pathogen and resident microflora. Samples like urine and swabs will have low target pathogen cell counts making it difficult to extract a high concentration of genomic DNA (gDNA). In contrast, human stool samples are comprised of a complex matrix of fibers, enzymes, undigested parts, and other inhibitors which have to be removed during DNA extraction (36). Both stool and sputum samples will need to be liquefied using a tissuelyser or decongestion solution, respectively (37). Fortunately, in the age of PCR based diagnostics, there are a wide array of methods and molecular kits for extracting DNA from clinical samples (37). Commercial kits for DNA extraction and purification, such as Qiagen's DNA Stool Mini Kit and QIAmp Microbiome DNA Kit can be adapted to metagenomics sequencing (38). However, these kits are designed for PCR analysis and will need to be combined with host DNA depletion and/or bacterial enrichment. Thus, while metagenomics has the ability to provide universal detection of pathogens, in reality specific nucleic acid extraction, strategies for target enrichment or host nucleic acid depletion will be needed for each pathogen or particular specimen type (34).

**Figure 1:**
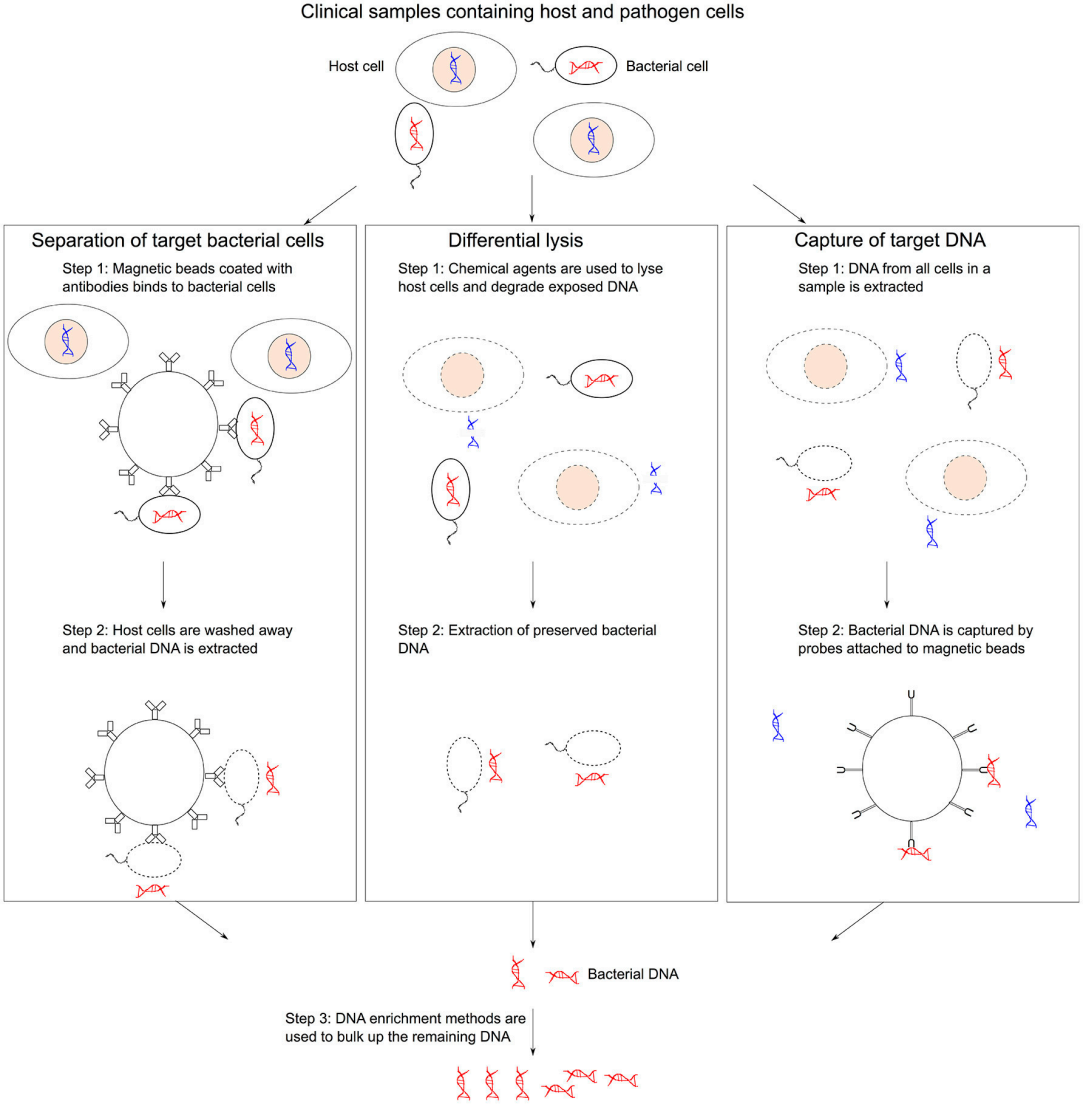
Methods of collecting bacterial gDNA and depleting host DNA. (1) Microbial separation involves pulling out bacterial cells using magnetic beads coated with antibodies from mixed samples followed by DNA extraction. (2) Differential lysis methods use selective agents to lysis host cells and then degrading the exposed host DNA before extracting bacterial DNA. (3) Targeted sequence capture approaches use magnetic beads that can hybridized to bacterial DNA to isolated specific sequences from a mixed sample post-DNA extraction. DNA enrichment methods can be used to bulk up the remaining DNA.

There are three key challenges of sequencing microbial organisms directly from a sample: (i) contamination of host DNA and other microorganisms, (ii) low cell abundance of the target organism present in the sample, and (iii) presence of DNA amplification inhibitors and other confounding variables in the clinical sample matrix. Therefore, several complementary methods have been developed to reduce the presence of host DNA or separate out the microbial DNA. Afterwards usually an enrichment method is then used to address the low microbial DNA. Noteworthy, the DNA enrichment process can increase the risk of selection bias. The key pre-treatment methods for metagenomics are summarized in Figure 1.

## Microbial Separation

Microbial cells can be separated from the sample matrix using chemical, physical, physicochemical or biological mechanisms (39). One of the established protocol is immunomagnetic separation (IMS), which has been successfully used to concentrate targeted bacteria and remove inhibitors to improve the quality of the extracted DNA for amplification-based methods (40, 41). IMS has since been applied to successful sequencing of *Chlamydia trachomatis* directly from genital swabs (42, 43). This approach uses magnetic beads coated with antibodies specific to chlamydial LPS to bind and extract chlamydial cells in clinical samples from the site of infection. The DNA extraction is then performed on the isolated cells followed by DNA purification. As clinical samples often do not carry sufficient quantities of target bacteria to give enough DNA for genome sequencing, IMS has been combined with multiple displacement amplification (MDA) to amplify DNA using the Φ29 polymerase and random hexamer primers (44). The combined IMS-MDA protocol has provided high-quality genomic DNA for *C. trachomatis* directly from clinical samples (42, 43). However, it must be noted that the clinical specimens used in these studies were urine and cervical swabs. Both sample types contained minimal human and microbiome DNA reducing non-specific binding of cells to the antibodies. The application of IMS-MDA protocols to rectal swabs in order to support direct sequencing of sexually transmitted pathogens in high-risk populations, such as men who have sex with men (MSM) could be less effective.

In addition to microbial separation of a targeted organism with antibodies, approaches have also been used to separate host DNA from microbial DNA allowing for an unbiased study of the microbiome within the specimen. For example, the NEBNext® Microbiome DNA Enrichment kit (New England Biolabs) selectively targets methylation sites in eukaryotic DNA and binds it to magnetic beads. This approach was used to directly sequence 13 *Neisseria gonorrheae* genomes directly from urine samples (32). Without enrichment, fewer than 1% of reads aligned to the *N. gonorrheae* reference genome, however, after using the NEBNext kit, the number of *N. gonorrheae* reads ranged from 2 to 43% of total number of sequenced reads. This provided sufficient coverage to obtain robust typing information for 11 of the 13 strains. While promising, this approach is still limited by the sample type (sterile vs. non-sterile site) and bacterial load as few reads were obtained for the pathogen of interest both in this example and from the CSF case mentioned previously (35). The success of this approach appear to be dependent on bacterial load and sample type.

## Depletion of Host Nucleic Acids

These methodologies focusing on depleting host cells from the original specimen by exploiting difference in cell surface structure between human cells and bacteria for selective lysis of host cells. Using a combination of selective cell lysis and DNase treatment, these techniques have had some success although high microbial load and a large original sample volume are often required (28, 45). One of the first applications of clinical metagenomics study to directly sequence *Mycobacterium tuberculosis* from respiratory specimens was based on osmotic lysis, where a large volume of sterile water is added to a sputum sample to increase the osmotic pressure and cause human cells to burst while leaving the more robust *M. tuberculosis* cells intact (45). A DNase enzyme was then used to degrade the liberated human DNA prior to *M. tuberculosis* DNA extraction. However, this approach will be severely limited for acquiring bacterial DNA from low load samples without enrichment (45). Another limitation is that some pathogens of interest, such as Gram-negative bacteria, are also susceptible to osmotic pressure.

A number of commercial kits are available for selective lysis of human or eukaryotic cells. Molzym's MolYsis Basic kit was used for direct sequencing of *M. tuberculosis* with greater efficacy than osmotic lysis only. The MolYsis kit uses a chaotropic buffer that selectively lyse human cells while keeping the bacterial cells intact (28). From this study sufficient DNA was obtained for antibiotic susceptibility prediction in 62% of samples. A critical advantage in this study was that genome sequencing was performed on a MinION platform (Oxford Nanopore), which has the ability to continue sequencing until sufficient coverage is obtained. Therefore, success rate can be lower when other, especially short-read sequencing, platforms are used because of static read coverage per sample. A comparison study on synovial fluid spiked with *Staphylococcus aureus* revealed that use of the MolYsis Kit produced a higher fold of microbial reads compared to the NEBNext® Microbiome DNA Enrichment kit (46).

Another study demonstrated an improved method of human cell depletion over the Molysis kit (47). The method sought to rely on mild detergents that are typically used to permeabilize mammalian cell lines for protein extraction or for the recovery of intracellular pathogens. Saponin, commonly used in hematology laboratories for hemolysis of human erythrocytes, was found to be the most effective in its differential effect on human vs. pathogen DNA in spiked cerebrospinal fluid (CSF) and nasopharyngeal aspirate specimens (40). In combination with Nanopore sequencing, saponin depletion can rapidly and accurately characterize the bacterial composition of the lower respiratory tract and provide antibiotic resistance data (48). This study showed that the saponin differential lysis method resulted in a 10^3^ fold decrease in host DNA in bronchoalveolar lavage and endotracheal aspirates. This depletion efficiency depends on using both a high concentration of saponin (2.2–2.5%) and salt buffer (5.5 M NaCl). Nanopore sequencing also has the significant advantage of providing real time data acquisition and analysis compared to the Illumina platform, allowing for fast turnaround time of clinical results.

## Targeted Enrichment of Pathogen DNA After Extraction

The targeted enrichment of pathogen DNA commonly involves hybridization of complement probes to the target bacterial genome. Agilent Technologies developed one such kit (SureSelect^XT^) originally for the enrichment of specific regions of large eukaryotic genomes for deep sequencing of a selected subset of genes (49, 50). However, it has since been adapted for deep sequencing of viral genomes directly from clinical samples (51, 52). This sequence capture method involves designing customized biotin-labeled RNA probes that can hybridized to a complete target genome sequence so that magnetic beads coated with the biotin-binding protein, streptavidin, can be used to extract the DNA (53). This approach can be suitable for culture-independent genome sequencing of *Chlamydia* species because strains within a species have highly conserved genome sequences with >90% nucleotide similarity. Therefore, probes can be designed using publicly available reference genomes to reliably capture target DNA for strains within the species. This has been successfully achieved for the identification of *C. trachomatis* in clinical swabs from patients with urethritis (31). Since then this approach has been applied to other *Chlamydia* species, including zoonotic pathogens *C. pecorum* and *C. psittaci* (54, 55). However, since the probe design depends on a reference sequence, this approach can be problematic when extended to pathogens with highly mosaic genomes, like *Salmonella* or *Escherichia coli*. Significant features in these pathogens, such as antibiotic resistance or virulence are often associated with mobile elements and genomic islands. Secondly, this approach may not be applicable for pathogen discovery as the emergent pathogen may not be captured and enriched by the probes.

Target enrichment has also been applied to the clinical situation for Tuberculosis diagnostics. In one case a clinical isolate from a patient with pulmonary tuberculosis was reported as resistant to rifampicin and low-level resistant to isoniazid based on initial results from Xpert MTB/RIF and Hain line probe assays (GenoType MDRTBplus v1.0), which target only *rpoB* and *inhA* genes in the *M. tuberculosis* genome (56). Therefore, the patient was placed on a combined drug regime including high-dose isoniazid. Metagenomic sequencing directly from the sputum sample identified a further *inhA* gene mutation that was consistent with high-level resistance to isoniazid and confirmed the absence of fluoroquinolone resistance. These findings informed treatment decisions with a shorten turnaround time by 12–14 days compared to conventional culture-based techniques. This study used the enrichment technology, SureSelect^XT^ (Agilent Technologies) to capture and amplify mycobacterial DNA (57), which remains prohibitively expensive for use as part of routine diagnosis. In addition, this method requires equipment not commonly found in most diagnostic laboratories, such as focused ultrasonicators for DNA shearing. However, the cost of the enrichment can be reduced by only targeting regions that encode drug resistance (29).

## Challenges of Implementing Metagenomics to Public Health

Risk of laboratory contamination and enrichment biases are of particular concern as the use of highly sensitive clinical metagenomics becomes more widespread. Possible sources of DNA contamination include extraction reagents and columns, PCR reagents and library preparation reagents (58, 59). Another concern is background DNA contained within a WGS library preparation laboratory, particularly a laboratory routinely performing high-throughput sequencing on cultured isolates. Identification of pathogens that are routinely sequenced after cultivation in a clinical sample should be scrutinized as it may indicate sample contamination. However, this can addressed by adequate quality control measures, such as inclusion of negative “no-template” controls for each preparation step, as part of every sequencing run, to monitor for contamination by commensal DNA (34).

Two significant objectives must be achieved to facilitate the implementation of metagenomics into public health laboratory surveillance and outbreak investigations. Firstly, the analytical sensitivity of metagenomic methods should be improved and validated (34). This evaluation should include testing of reproducibility and repeatability of testing which might require involvement of multiple laboratories. A key added value of metagenomics is likely to be its effectiveness on samples collected in earlier, most transmissible, stages of infectious disease. Secondly, the cost of consumables per sample should decrease significantly to support the uptake of this technology. Currently, DNA enrichment methods remain the most expensive aspect of metagenomic testing, with estimated cost up to $350 USD per sample (57).

## Conclusion

Metagenomics has yet to be implemented into routine diagnosis and few long term studies have been conducted. However, future advancements in sequencing technology will most likely be the deciding factor as to when metagenomics will be integrated into public health surveillance. Sensitivity of shotgun metagenomics will improve as new sequencing platforms are released that can efficiently generate longer sequencing reads with higher depths. In addition due to the various sample types and data analysis methods available, laboratories will need to clearly define the intended clinical usage and range of pathogens that will be detected as this will greatly impact the choice of methodology for sample preparation and DNA extraction.

## Author Contributions

NB and RR complied and wrote the manuscript. VT and VS were involved in review and editing.

### Conflict of Interest Statement

The authors declare that the research was conducted in the absence of any commercial or financial relationships that could be construed as a potential conflict of interest.
